# Relationship between oral and gut microbiota in elderly people

**DOI:** 10.1002/iid3.266

**Published:** 2019-07-15

**Authors:** Megumi Iwauchi, Ayako Horigome, Kentaro Ishikawa, Aya Mikuni, Manabu Nakano, Jin‐zhong Xiao, Toshitaka Odamaki, Shouji Hironaka

**Affiliations:** ^1^ Department of Special Needs Dentistry, Division of Hygiene and Oral Health Showa University School of Dentistry Tokyo Japan; ^2^ R&D Division, Next Generation Science Institute Morinaga Milk Industry Co, Ltd Kanagawa Japan; ^3^ R&D Division, Food Ingredients & Technology Institute Morinaga Milk Industry Co, Ltd Kanagawa Japan

**Keywords:** bacterial transition, elderly, fecal microbiota, oral microbiota

## Abstract

**Aim:**

Recent studies have suggested that oral bacteria induce systemic inflammation through the alteration of gut microbiota. We examined the relationship between oral and gut microbiota to evaluate the transition of oral bacteria to the gastrointestinal tract.

**Methods:**

Oral samples from subgingival plaque and tongue‐coating and fecal samples were collected from 29 elderly subjects (age, 80.2 ± 9.1 years) and 30 adults (age, 35.9 ± 5.0 years). Genomic DNA was extracted from all samples, and DNA sequencing of bacterial 16S rRNA genes was performed for microbiota analysis. UniFrac distances were calculated to evaluate the similarity between microbial communities.

**Results:**

Unweighted UniFrac distance indicated that the elderly group had a higher similarity between fecal and subgingival plaque microbiota than the adult group. Indeed, some bacterial taxa found in oral samples had a significantly higher prevalence in the feces of the elderly group than in that of the adult group.

**Conclusions:**

The prevalence of oral bacterial transition to gut may be higher in the elderly than in adults, expecting that oral health care in the elderly will affect their gut microbiota composition and consequently promote human health.

**ABbreviations**
BOP: bleeding on probing
CPI 2013: Community Periodontal Index 2013
OTUs: operational taxonomic units
PBS: phosphate‐buffered saline
PCoA: principal coordinate analysis
PCR: polymerase chain reaction
PPD: probing pocket depth

## 1 INTRODUCTION

The composition of human gut microbiota changes with aging.^1^, ^2^ *Actinobacteria*, including the genus *Bifidobacterium*, which has been reported to downregulate proinflammatory responses,^3^, ^4^ decreases with age, whereas *Proteobacteria*, to which many inflammation‐inducing bacteria belong, increases with age.^2^ Recent evidence suggest that the age‐related gut dysbiosis in elderly people leads to a low‐grade chronic inflammatory state that could be linked to most of the age‐related health problems, such as dementia, Alzheimer's disease, type 2 diabetes, cancer, and atherosclerosis.^2^, ^5^, ^6^

Oral bacteria also are known to be related to various diseases. Some epidemiologic studies have shown that periodontal disease, which is caused by periodontopathic bacteria, such as *Porphyromonas gingivalis*, *Tannerella forsythia*, and *Treponema denticola*, is a risk factor for a variety of diseases, such as atherosclerotic vascular disease,^7^ type 2 diabetes,^8^ and nonalcoholic fatty liver disease.^9^ Because we swallow approximately 600 mL of saliva per day containing up to 10^9^ bacteria/mL^10^, it is reasonable that certain oral bacteria are suspected to be inducers of various diseases through disturbance of gut microbiota. Others have reported that repeated oral administration of *P. gingivalis* disturbed the gut microbiota composition, thereby inducing systemic inflammation in mice.^11^, ^12^, ^13^ A recent comparative genome analysis showed no difference between oral‐ and gut‐derived *Fusobacterium* strains in patients with colorectal cancer.^14^ These evidence also indicated the importance of the oral microbiome as an inducer or enhancer for several systemic diseases, such as type 2 diabetes,^11^ arthritis,^13^ and colorectal cancer.^14^

Several antibacterial factors exist in the gastrointestinal tract, such as gastric acid and bile acid. Considering the decline of gastrointestinal tract functionality in the elderly, it is possible that more prevalent transition of oral bacteria to the gastrointestinal tract occurs in the elderly than in healthy adults. Indeed, the compositional rate of oral bacteria, such as *Porphyromonas*, *Fusobacterium*, and *Pseudoramibacter*, have been reported to increase in the gut with age.^2^ However, little is known about the detail of bacterial transition from oral to gut environment in the elderly subject.

We evaluate whether the transition of oral bacteria to the gastrointestinal tract is more prevalent in the elderly than in adults. We conducted a 16S rRNA gene analysis on microbiota of fecal, subgingival plaque, and tongue‐coating samples collected from elderly and healthy adults.

## 2 MATERIALS AND METHODS

### 2.1 Subjects recruitment

Residents in two nursing homes and healthy adult volunteers were recruited from January to March 2017. Participants were screened based on the exclusion criteria after providing written informed consent from subjects or their relatives. Exclusion criteria included having difficulty obtaining a dental examination, having difficulty in sampling, remaining number of teeth (<10), having received treatment for periodontitis in the past 1 month, receiving treatment for a chronic disease (for healthy adults), and past history of critical illness (for healthy adults). The sample size was set at 30, which was the maximum possible entry number during the study period. The 60 participants (30 elderly individuals who required nursing care [elderly] and 30 adults) who were recruited and clinically investigated were instructed to fill out a questionnaire on sex, age, frequency of brushing teeth, and use of dentures.

The study was done in accordance with the tenets of the Declaration of Helsinki 1975 and as revised in 2013. The study design was reviewed and approved by the research ethics committee of the School of Dentistry, Showa University (no. 2016‐005).

### 2.2 Dental examination

Oral assessments, including number of teeth, bleeding on probing (BOP), and probing pocket depth (PPD), were performed by three trained and calibrated dentists. On the basis of the BOP and PPD results, periodontal disease progression status was evaluated using Community Periodontal Index 2013 (CPI 2013).

### 2.3 Oral sampling and bacteriologic assessment

After oral assessments, oral samples were collected 1 hour or later after a meal or oral care, such as tooth brushing. The tongue coating was collected from a 2 cm^2^ area in the tongue center using a sterile swab with constant pressure. Subgingival plaque was obtained from the cervical region on the buccal side of the teeth that had the maximum value on PPD examination using a paper point. The swab and the paper point were suspended in 1 mL sterile saline and 1 mL phosphate‐buffered saline (PBS) stored in vials,^15^ respectively, and the total number of bacteria and the number of *P. gingivalis* were measured using the polymerase chain reaction (PCR)‐invader method.^16^ Detection limits of total bacteria and *P. gingivalis* were 1000 copies/10 μL and 10 copies/10 μL saline/PBS, respectively. Bacterial counts were determined as log_10_ copies/10 μL saline/PBS among individuals over detection limits.

### 2.4 Fecal sampling

Fecal sampling was conducted within a week after the oral sampling. For the elderly, fecal samples were collected from the subject's diaper by staff in the nursing home; for adults, the samples were collected from the toilet bowl by subjects themselves. All samples were immediately frozen and stored at below −18°C until delivering to the laboratory. Immediately upon receipt, the fecal samples were stored at −80°C until further analysis.

### 2.5 Microbiota analysis

Microbiota analysis was performed as described previously^2^ with minor modifications. Briefly, DNA extraction from fecal samples, PCR amplification, and DNA sequencing of the V3‐V4 region of the bacterial 16S rRNA gene by an Illumina MiSeq instrument (Illumina, San Diego, CA) was performed as described previously.^17^ After removing sequences consistent with data from the Genome Reference Consortium human build 38 (GRCh38) or PhiX 174 from the raw Illumina paired‐end reads using the bowtie‐2 program^18^ (ver. 2‐2.2.4), the 3′ region of each read with a PHRED quality score of less than 17 was trimmed. Trimmed reads of less than 150‐base pairs (bp) long, with an average quality score of less than 25 or those lacking paired reads also were removed. The trimmed paired‐end reads were combined using the fastq‐join script in EA‐Utils (ver. 1.1.2–537; Aronesty E. Comparison of sequencing utility programs. *Open Bioinforma J*. 2013;7:1–8). Potential chimeric sequences were removed by reference‐based chimera checking in USEARCH^19^ (ver. 5.2.236) and the gold database (available in the public domain at http://drive5.com/otupipe/gold.tz). The nonchimeric sequences were analyzed using the QIIME software package version 1.8.0.^20^ The sequences were assigned to operational taxonomic units (OTUs) using Open‐reference OTU picking^21^ with a 97% threshold of pairwise identity and subsequently classified taxonomically using the Greengenes reference database (available in the public domain at ftp://greengenes.microbio.me/greengenes_release/gg_13_5/gg_13_8_otus.tar.gz).^22^

### 2.6 Evaluation of the similarity between microbial communities

The similarity of microbial communities between subjects or samples was evaluated based on UniFrac distance. Principal coordinate analysis (PCoA) based on unweighted (based on presence or absence of observed bacterial taxa) and weighted (based on the abundance of observed bacterial taxa) UniFrac distances were performed using QIIME version 1.8.0 software. Closer plots in the PCoA figure indicate more similar microbiota composition.

### 2.7 Statistical analysis

Statistical analyses were conducted using the IBM SPSS Statistics, version 22.0, statistical software package (IBM, Armonk, NY). Intergroup differences in the subject background were analyzed using the *χ* ^2^ test or Fisher exact test for categorical data and the unpaired Student *t* test for measured variables. Intergroup differences of bacterial counts were analyzed on bacterial counts after logarithmic transformation, by substituting data with log_10_ 10 values for individuals under the detection limits of *P. gingivalis*. Intergroup differences in UniFrac distance were assessed by Mann‐Whitney *U* test and Kruskal‐Wallis and the post‐hoc Dunn multiple‐comparisons tests. The *χ* ^2^ test or Fisher exact test were used to assess intergroup differences in the detection rate of oral bacteria in feces.

### 2.8 Data deposition

DNA sequences corresponding to the 16S rRNA gene data have been deposited in DNA Data Bank of Japan (DDBJ) under accession number DRA008582.

## 3 RESULTS

### 3.1 Subjects

A total of 60 participants (30 elderly and 30 adults) were enrolled in this study. An elderly subject dropped out owing to rejection of fecal sampling. Table 1 shows the characteristics of subjects in each group. The ratio of females was significantly higher in the elderly than in the adult groups. However, no obvious difference in oral and fecal microbiota was observed between male and female subjects (Figure S1), suggesting that the sex imbalance did not affect the study results. CPI 2013 showed a more severe inflammatory condition of periodontal status in the elderly than in the adult groups. In agreement with CPI 2013 scores, the cell number of *P. gingivalis* in the oral samples was significantly higher in the elderly than in the adult groups. On the contrary, the total number of tongue‐coating bacteria was significantly higher in the adult than in the elderly groups.

**Table 1:** Subject background

	Adult		Elderly		*P* value
Number of subjects	30		29		
Male/Female	18/12		7/22		.012^a^, *
Age, y	35.9 ± 5.0		80.2 ± 9.1		<.001^b^, **
Frequency of brush teeth, per d	2.3 ± 0.7		2.4 ± 0.5		.479^b^
Number of teeth	28.5 ± 1.5		18.8 ± 6.2		<.001^b^, **
Community Periodontal Index 2013					
Gingival bleeding scores					
0	16		5		.006^a^, **
1	14		24		
Pocket scores					
0	3		5		.246^a^
1	27		22		
2	0		2		
Total bacterial count (prevalence)					
Tongue coating	6.27 ± 0.40	(100)	6.02 ± 0.44	(100)	.027^b^, *
Subgingival plaque	3.60 ± 0.63	(100)	4.13 ± 0.64	(100)	.002^b^, **
Bacterial count (prevalence) of *P. gingivalis*					
Tongue coating	1.93 ± 0.81	(6.67)	2.25 ± 0.71	(58.6)	<.001^b^, **
Subgingival plaque	2.24 ± 1.08	(6.67)	2.74 ± 0.90	(34.5)	.010^b^, **

*Note*: Measured variable data are expressed as mean ± SD.

^a^ Intergroup differences were analyzed using the *χ* ^2^ or Fisher test.

^b^ Intergroup differences were analyzed using unpaired Student *t* test.

* *P* < .05.

** *P* < .01.

### 3.2 Overview of fecal and oral microbiota composition

A total of 177 feces, subgingival plaque, and tongue‐coating samples were collected from 59 subjects. A total of 2 869 516 high‐quality paired sequences were obtained from the 177 samples, with 16 031 ± 3009 reads per sample.

**Figure 1:**
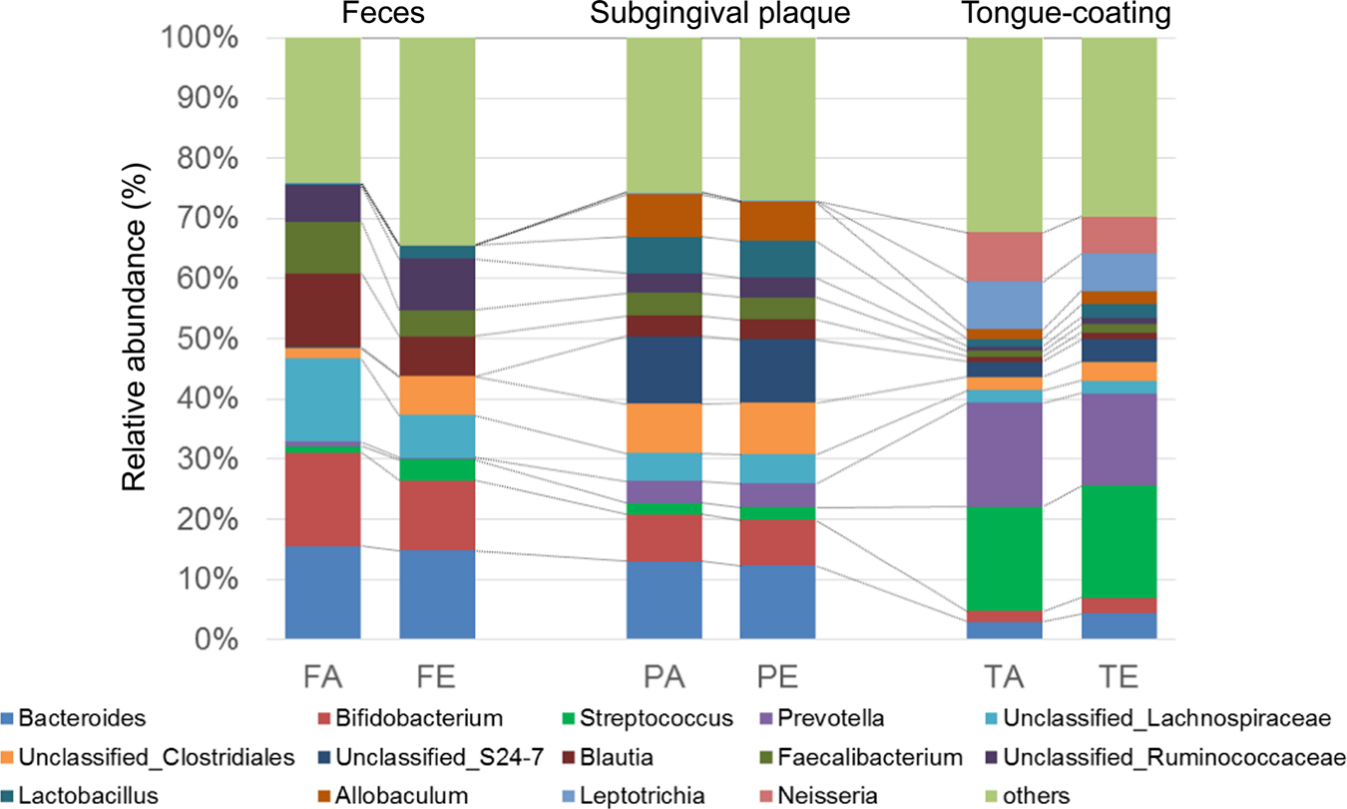
Compositions of each microbiota at the genus level. Labels except for “others” indicate the genera at average relative abundance (≥5%) in at least one sampling site. FA, feces of adult; FE, feces of elderly; PA, subgingival plaque of adult; PE, subgingival plaque of elderly; TA, tongue coating of adult; TE, tongue coating of elderly

Figure 1 shows the compositions of each microbiota at the genus level. The compositions apparently were different among feces, subgingival plaque, and tongue‐coating microbiota.

**Figure 2:**
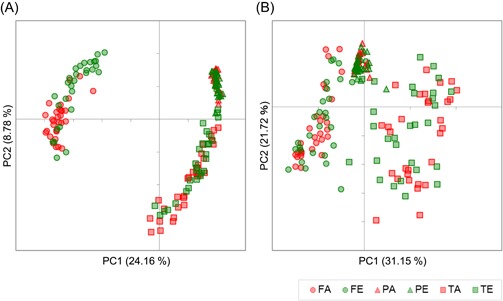
Principal Coordinate Analysis (PCoA) of fecal and oral microbiota. A, Unweighted and (B) weighted UniFrac PCoA of fecal, subgingival plaque and tongue‐coating microbiota in samples collected from the healthy adult (n = 30) and elderly subjects (n =* *29). Unweighted and weighted distances are calculated based on the presence or absence and the relative abundance of observed bacterial taxa, respectively. Closer plots in the PCoA figure indicate more similar microbiota composition. The percentage of variation explained by principle coordinates (PC) is indicated on the axes. FA, feces of adult; FE, feces of elderly; PA, subgingival plaque of adult; PE, subgingival plaque of elderly; TA, tongue coating of adult; TE, tongue coating of elderly

Then, we evaluated the extent of similarity between microbial communities using UniFrac distances analysis. Figure 2 shows PCoA plots based on UniFrac distances. The plots of feces, subgingival plaque, and tongue‐coating microbiota were clustered respectively. Particularly, unweighted PCoA plots (Figure 2A) indicated a clear separation between fecal and oral samples compared to weighted PCoA plots (Figure 2B).

### 3.3 Similarity difference of fecal and oral microbiota between elderly and adult

**Figure 3:**
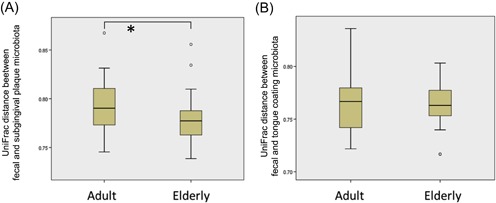
Unweighted UniFrac distance between fecal and subgingival plaque microbiota (A) and between fecal and tongue‐coating microbiota (B) compared between the adult and elderly groups. *P < .05

We compared the similarity of fecal and oral microbiota between elderly and adults. Unweighted UniFrac distance between the fecal and subgingival plaque microbiota in the elderly group was significantly shorter than that in the adult group (Figure 3A).

We then performed further investigation to compare the bacterial taxa detected in oral and fecal samples between the elderly and adults. A total of 132 taxa detected in subgingival plaque or tongue‐coating microbiota were found in the corresponding fecal samples. Of these taxa, 14 detected in the subgingival plaque sample had a significantly higher prevalence in the feces of the elderly than of the adult groups (Table 2). Other 14 taxa detected in the tongue‐coating sample also had a significantly higher prevalence in the feces of the elderly group. On the other hand, only three oral taxa had a significantly higher prevalence in the feces of the adults than in that of the elderly. Interestingly, not all taxa have been registered as oral bacteria in Human Oral Microbiome Database^23^.

**Table 2:** Detection rate of subgingival plaque and tongue‐coating bacteria in feces

	Taxon including oral species^a^	Detected in both feces and subgingival plaque (%)			Detected in both feces and tongue coating (%)		
		Adult	Elderly	P value	Adult	Elderly	P value
*Bacterial taxa significantly higher in elderly*							
*Eggerthella*	No	63.3	72.4	.64	6.7	41.4	<.01**
*Corynebacterium*	Yes	0.0	13.8	.05	0.0	31.0	<.01**
*Butyricimonas*	No	36.7	48.3	.52	3.3	31.0	<.01**
*YS2*;f	No	0.0	37.9	<.01**	0.0	13.8	.05
*Lactobacillales*;f	Yes	0.0	17.2	.02*	0.0	0.0	1.00
*Mogibacterium*	Yes	0.0	17.2	.02*	6.7	37.9	<.01**
*Christensenellaceae*;g	No	46.7	79.3	.02*	13.3	55.2	<.01**
*Dehalobacterium*	No	10.0	58.6	<.01**	0.0	34.5	<.01**
*SMB53*	No	6.7	20.7	.15	0.0	17.2	.02*
*Pseudoramibacter_Eubacterium*	Yes	30.0	20.7	.60	0.0	13.8	.05
*Peptococcaceae*;g	Yes	0.0	37.9	<.01**	0.0	24.1	<.01**
*Ruminococcaceae*; Other	Yes	36.7	69.0	.03*	23.3	48.3	.08
*Bulleidia*	Yes	3.3	20.7	.05	16.7	44.8	.04*
*RF32*;f	No	13.3	48.3	<.01**	6.7	24.1	.08
*Bilophila*	No	60.0	93.1	<.01**	43.3	72.4	.05*
*Desulfovibrio*	Yes	16.7	69.0	<.01**	13.3	65.5	<.01**
*Campylobacter*	Yes	0.0	27.6	<.01**	6.7	37.9	<.01**
*RF39*;f	No	3.3	58.6	<.01**	3.3	55.2	<.01**
*TM7‐3*;o	Yes	3.3	24.1	.03*	16.7	27.6	.49
*Akkermansia*	No	36.7	72.4	.01*	26.7	62.1	.01*
*Bacterial taxa significantly higher in adults*							
*Lactococcus*	Yes	76.7	48.3	.05*	76.7	48.3	.05*
*Megamonas*	No	26.7	0.0	<.01**	23.3	0.0	.01*
*Megasphaera*	Yes	46.7	10.3	<.01**	46.7	10.3	<.01**

*Note*: Intergroup differences were analyzed using the *χ* ^2^ or Fisher test.

^a^ Species registered as oral taxa in Human Oral Microbiome Database.

* *P* < .05.

** *P* < .01.

## 4 DISCUSSION

A vast amount of knowledge of the microbiota has been provided from research consortiums, such as the Human Microbiome Project (HMP)^24^ and Metagenomics of the Human Intestinal Tract (MetaHIT).^25^ These data open the door for implementation of human microbiota, and, in particular, oral and gut bacteria have been reported to be related to various systemic diseases.^2^, ^5^, ^6^, ^7^, ^8^, ^11^ Although some previous reports have suggested the relationship among oral bacteria, gut dysbiosis, and systemic diseases,^2^, ^13^, ^14^, ^15^ little is known about the detail information in humans. We focused on the relationship between oral and fecal microbiota, then investigated whether the transition of oral bacteria to the gastrointestinal tract was more prevalent in the elderly than in healthy adults.

Genus compositions of microbiota and UniFrac PCoA based on the microbiota compositions showed a distinctive microbiota profile at each sampling sites (Figures 1 and 2). Clear separation of fecal and oral microbiota indicated by unweighted UniFrac PCoA suggested that the bacterial members were greatly different between fecal and oral microbiota. Unlike the previous reports,^26^, ^27^ in this study, the subgingival plaque microbiota was observed to contain certain amount of *Bacteroides* and *Bifidobacterium*. We predict that these were oral species such as *Bacteroides heparinolyticus* and *Bifidobacterium dentium* and not the members of gut microbiota. Further high‐resolution analyses of microbiota are needed to validate this prediction.

The analysis based on unweighted UniFrac distance showed a higher similarity between the fecal and subgingival plaque microbiota in the elderly than in the adult groups (Figure 3A). This result suggested that the transition of subgingival plaque bacteria to the gut is more prevalent in the elderly than in adults. Furthermore, higher abundance of bacterial taxa, including *Bilophila*,^28^ *Desulfovibrio*,^29^ and *Campylobacter*,^30^ which have been reported as predictors or incidents of diseases, such as gastroenteritis and bacteremia, were shared by the fecal and subgingival plaque or the tongue‐coating microbiota more frequently in the elderly subjects (Table 2). On the contrary, only three bacterial taxa, which were commonly found in natural environment and animal gut, were shared by fecal and oral microbiota more frequently in the adult subjects. These results also supported our hypothesis that the transition of oral (subgingival plaque and tongue coating) bacteria to the gut is more prevalent in the elderly than in adults. Moreover, the bacterial taxa in the subgingival plaque and the tongue‐coating microbiota described in Table 2 were suggested to have transferred to the gastrointestinal tract in the elderly. Considering the decline in gastrointestinal tract functionality in elderly people, it could be possible that the oral bacteria effluxed from subgingival plaque and the tongue coating to saliva was swallowed to some extent, and reached the gastrointestinal tract with less extinction by gastric juice and/or bile acid compared to what occurred in healthy adults. Another possibility was that the subgingival plaque bacteria invaded the gingival tissue by inducing periodontal inflammation, then disseminated into the systemic circulation to reach the large intestine. Indeed, Abed et al^31^ reported that *Fusobacterium* used a hematogenous route to reach the large intestine. A significantly higher number of total bacteria in the elderly subgingival plaque also might contribute to the higher prevalence of bacterial transition from the mouth to the gastrointestinal tract.

The prevalence of predictable transited bacteria from entrance to exit of our digestive system calculated in this study was lower than we expected from our previous cross‐sectional study.^2^ The most plausible reason is that the oral environment of the elderly subjects enrolled in this study has been kept clean by caregivers in the nursing home. Also, we failed to detect the relationships of the similarity between fecal and oral microbiota with oral or systemic conditions, such as usage of dentures, medication, and diseases. These are the limitation of our study. A large‐scale interventional trial will be necessary to reveal whether an elaborate oral health care could change the transition of oral bacteria to the gut and reduce the age‐related health problems.

In conclusion, our results suggested that a higher prevalence of oral bacterial transition to the gut in the elderly than in adults. It is expected that the possibility of promotion of human health by proper oral health care will be defined in the future.

## CONFLICT OF INTERESTS

Four of the authors, A Horigome, M Nakano, JZ Xiao, and T Odamaki, are employees of Morinaga Milk Industry. The other authors declare no conflict of interests.

## Supporting information

Supplementary information

Supplementary information
