# Supplementary figures and images for: Relationship between oral and gut microbiota in elderly people
Source: Immun Inflamm Dis. 2019 Jul 15;7(3):229–36. doi: 10.1002/iid3.266 (PMC6688080; doi:10.1002/iid3.266)

**Figure S1 Difference of fecal and oral microbiota between male and female subjects**

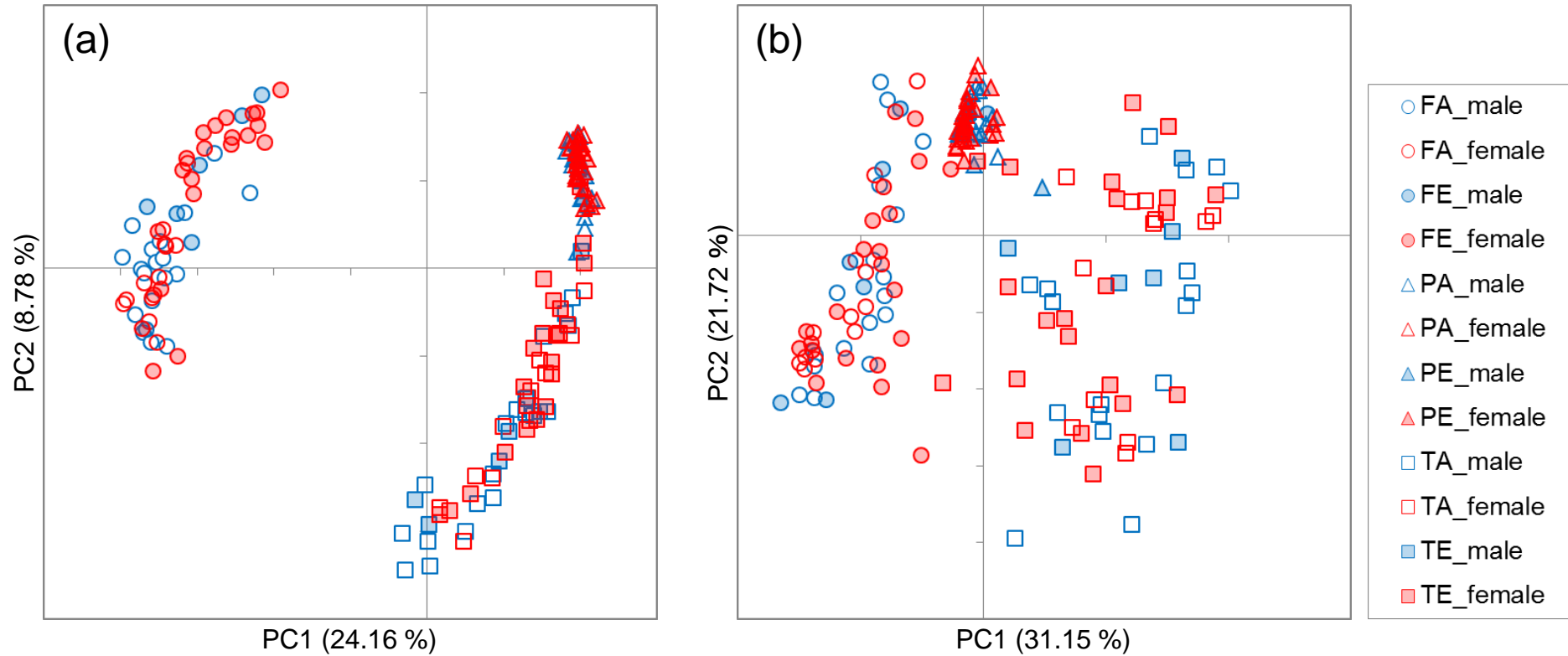

Supplement: Supplementary file 1 — Supplementary information [file IID3-7-229-s001.pdf]
