# Supplementary material for: Relationship between oral and gut microbiota in elderly people
Source: Immun Inflamm Dis. 2019 Jul 15;7(3):229–36. doi: 10.1002/iid3.266 (PMC6688080; doi:10.1002/iid3.266)
Supplement: Supplementary file 2 — Supplementary information [file IID3-7-229-s002.docx]

**Supporting Information**

**Supplemental Figure S1.** Difference in fecal and oral microbiota between male and female subjects. (**a**) Unweighted and (**b**) weighted UniFrac PCoA colored *blue* (male) and *red* (female) using same data in Figures 2a and 2b. Unweighted and weighted distances are calculated based on the presence or absence and the relative abundance of observed bacterial taxa, respectively. Closer plots in the PCoA figure indicate more similar microbiota composition. The percentage of variation explained by principle coordinates (PC) is indicated on the axes. FA, feces of adult; FE, feces of elderly; PA, subgingival plaque of adult; PE, subgingival plaque of elderly; TA, tongue-coating of adult; TE, tongue-coating of elderly
